# Evaluating the effectiveness of low-level laser therapy in patients undergoing lower third molar extraction: A double-blinded randomized controlled trial

**DOI:** 10.4317/medoral.26894

**Published:** 2024-11-25

**Authors:** Marcelo José Uzeda, Alice Maria de Oliveira Silva, Lavínia Nascimento da Costa, Felipe Santos de Brito, Gustavo Vicentis Oliveira Fernandes, Rodrigo Figueiredo Resende

**Affiliations:** 1 PhD. Department of Oral and Maxillofacial Surgery, Stricto Sensu Postgraduate Program in Dentistry, Iguaçu University, Rio de Janeiro, Brazil; 2 PhD. Department of Oral Surgery, Fluminense Federal University, Rio de Janeiro, Brazil; 3 M.Sc.. Oral and Maxillofacial Surgery and Traumatology at Universidade Federal Fluminense, Rio de Janeiro, Brazil; 4 MSc. Clinical Dentistry at Iguaçu University, Rio de Janeiro, Brazil; 5 PhD. A. T. Still University - Missouri School of Dentistry and Oral Health, St. Louis, MO, U.S.A; 6 Centre for Interdisciplinary Research in Health (CIIS), Universidade Católica Portuguesa, Viseu, Portugal

## Abstract

**Background:**

The use of low-level laser is a therapeutic resource that has been widely used in medicine in general, which has properties capable of modulating inflammatory effects such as pain, edema, and trismus resulting from oral surgeries for the extraction of impacted third molars. This study aimed to evaluate the effect of laser therapy at two different wave frequencies (660nm and 808nm) in patients undergoing impacted 3rd third lower molar extraction. For this, 30 patients were selected and randomly distributed into three groups with 10 individuals each (Control Group, 660nm Group, and 808nm Group).

**Material and Methods:**

Each participant, according to their experimental group, received irradiation before the start of each surgery and immediately after the surgical procedure. The data regarding edema, pain intensity, and trismus collected before the surgeries, immediately after, and after seven days were spreadsheeted and analyzed to determine the mean and standard deviation. After the Shapiro-Wilk normality test, differences between the experimental groups were determined using Multiple Analyses of Variance, considering a significance level of 5% (*p*<0.05).

**Results:**

The results showed no statistically significant difference between the experimental groups in the analyzed items. Despite this, both groups irradiated with 660nm and 808nm frequencies experienced a reduction in the perception of postoperative pain, thus suggesting the benefit of using low-power laser therapy as an adjunct in the surgical treatment of impacted lower third molars.

**Conclusions:**

Despite the results showing no differences between the groups, using LLLT resulted in lower pain perception in the irradiated participants compared to those in the control group. This fact supports the use of LLLT as an adjuvant therapy in patients undergoing oral surgery procedures to remove impacted third molars.

** Key words:**Third molar, impacted teeth, low-level laser therapy, inflammation, extraction, dentistry, oral surgery.

## Introduction

Extraction procedures for third molars are the most common surgical oral and maxillofacial surgery (1-4). The postoperative period is typically accompanied by inflammatory effects that are pretty uncomforTable for the patient (5-8), such as increased volume (edema), limited mouth opening (trismus), and pain (9). This reaction, caused by the intense inflammatory reaction within the first three days after the surgical procedure, compromises patients' quality of life during the healing period (10,11). The anatomical region where the surgical wound is located is constantly irritated by chewing and diction movements; in addition, it is challenging to access plaque control methods. These factors contribute to more significant postoperative discomfort for the patient (12). Moreover, these events cause changes in the patients' routines, which may negatively influence them, if necessary, future surgeries (1).

After any surgical procedure, a typical response to the injuries caused involves three overlapping and distinct stages: (A) hemostasis and inflammation, (B) new tissue formation, and (C) remodeling (13). These steps were well-demonstrated by Kahn *et al*. (14), who suggested a microsurgical approach level to reduce possible trauma and, consequently, the inflammatory profile. Therefore, pain generally reaches its peak within 5 hours after the intervention when the effects of the local anesthetic cease, while edema appears in its most advanced stage after approximately 48 hours (1,3-5). Furthermore, limited mouth opening is another factor that makes it difficult to clean the operated area and feed the patient, thus worsening the postoperative clinical condition.

Several therapeutic methods have been used to minimize the postoperative effects resulting from surgeries to extract impacted third molars, such as the use of analgesic and anti-inflammatory drugs, cryotherapy, compression therapy, and laser therapy, among others; without however, there is a consensus on the most effective method (1,2). Recent studies on the use of Low-Level Laser Therapy (LLLT) have demonstrated the modulatory capacity over the inflammatory process without promoting adverse effects, reducing pain and edema and promoting the repair of damaged tissues through the reduction of vascular permeability, making infiltration of neutrophils and reducing the presence of inflammatory enzymes and cytokines (6,7,15).

Thus, the goal of this double-blinded randomized controlled trial was to evaluate the adjuvant effects of using LLLT in patients undergoing surgery for the extraction of impacted third molars, seeking to establish a safe and effective therapeutic protocol that offers greater postoperative comfort and well-being.

## Material and Methods

This study was supported by the Fundação Carlos Chagas Filho de Amparo à Pesquisa do Estado do Rio de Janeiro (FAPERJ, Brazil) and was carried out following the guidelines and regulatory standards for research involving human beings set out in Normative Resolution no. 466/2012 from the National Health Council (CNS, Brazil), Declaration of Helsinki (updated in 2013), and was approved by the Research Ethics Committee of the Iguaçu University (RJ, Brazil) (no. 31.91.123).

The quality assessment of this study was carried out based on the CONSORT statement. All research participants were previously informed of the criteria necessary for participation in this project and signed the informed consent form. Thus, the voluntary research participants were selected from the Oral Surgery Clinic of the Faculty of Dentistry of the Iguaçu University and the Federal Fluminense University, following the pre-established inclusion and exclusion criteria.

- Eligibility criteria

The inclusion criteria were: (a) patients aged between 18 and 30; (b) any gender; (c) healthy; (d) presenting a mesioangular lower 3rd molar with class 1 or 2 impactions, A or B. The exclusion criteria were: (a) systemic diseases, even controlled; (b) neurological or psychiatric disorders; (c) routine use of anti-inflammatory drugs; (d) history of photosensitivity; (e) acute pericoronitis; (f) advanced periodontal disease; (g) pregnant women, infants; and (h) with any uncontrolled systemic disease.

- Sample size and Randomization process

The sample size calculation was developed following the use of low-level laser therapy (LLLT) in patients undergoing lower third molar extraction (16), observing the comparison of the results for the primary symptom of the patient (pain). The study presented, respectively, averages for the laser and placebo groups of 2.901 ± 0.453 and 4.324 ± 2.67. Considering power (1-, where is the risk of type II error) of 95%, of 0.05, and alpha of 0.001, the number of patients per group was 10; totaling 30 patients.

After the clinical and radiographic examination, the diagnosis for extracting the 3rd molar based on Winter’s and Pell and Gregory’s classifications were confirmed by two professionals (MJU and RFR), individually, who were expert professors in Oral Surgery. Volunteers (blinded) participating in the research were randomly divided by a different and blinded author (LNC), using the envelope method, who distributed the patients into three distinct groups: Group C (Control), Group 1 (LLLT-660nm), and Group 2 (LLLT-808nm).

- Surgical protocol

The participants underwent extraction of unilaterally impacted third molars under local anesthesia with 4% articaine and 1:100,000 epinephrine, respecting the maximum dose/kg (Nova DFL, Rio de Janeiro, Brazil). All the procedures were developed by the same professional (MJU). Then, incisions with a scalpel blade n.15 were made, followed by subperiosteal detachment with a Molt-type detacher to allow exposure to the respective dental elements. Total flaps (periosteal and mucosa detachments) were performed buccally, allowing the preservation of the anatomical structures of the lingual site. Ostectomies and teeth sections were performed with a Zekrya bur under abundant and constant irrigation of 0.9% saline solution.

After extraction, the alveoli were gently explored, washed with 0.9% saline solution, and sutured with 4.0 silk thread (J&J Ethicon®, São Paulo, Brazil). All participants received a prescription of Nimesulide 100mg (EMS, São Paulo, Brazil) every 12 hours during the first two postoperative days and analgesia with 750 mg of paracetamol (Tylenol®, Janssen-Cilag, São Paulo, Brazil) every 6 hours during the first 24 hours. In addition, they were also instructed to perform oral hygiene using 0.2% Chlorhexidine gel twice a day, starting on the day of surgery and maintaining it for 14 days.

- Irradiation protocol

Participants of Group 1 (LLLT-660nm) and Group 2 (LLLT-808nm) were irradiated with LLLT at six intra-oral points for 20 seconds each (mesio-buccal, disto-buccal, mesio-lingual, disto-lingual, mesio-occlusal, and disto-occlusal) and two extra-oral points (anterior and posterior edges of the masseter muscle, 1 cm above the lower edge of the mandible) before the surgery and immediately after the procedure. Participants in the control group were not irradiated. After seven days, the sutures were removed, and the final evaluation was carried out in all groups.

- Post-operative assessment

All postoperative assessments were carried out by two researchers who were different from those who had participated in the previous stages (AMOS and LNC). Three main parameters were evaluated: (A) pain, (B) edema, and (C) mouth opening. Pain intensity was assessed using a Visual Analogue Scale (VAS), which consisted of a linear scale measuring 10 cm in length, where 0 is equivalent to no pain and 10 is unbearable pain (1,2,5,6). One point was marked as a reference in the VAS, indicating pain intensity during the respective experimental periods. To assess edema, a millimeter tape was used to measure the distance between four predetermined facial measurement points: from the Exocantium to the Gion and from the Tragus to the Pogonion. These measurements were taken before the surgeries, immediately after, and on the 7th postoperative day (1,5,6,17). Mouth opening measurements were taken with a universal analog caliper (Western® - Etilux, São Paulo, Brazil) before, immediately after surgery, and after 7 days (1,5,6,17,18). All information collected was processed at the Clinical and Dental Research Laboratory at the Federal Fluminense University (LPCO-UFF, RJ, Brazil).

- Statistical analysis

The data obtained were organized in a spreadsheet (Excel®, Microsoft Office) and processed using Prism 8.0 software (GraphPad Software, Inc. La Jolla, CA, USA) to determine mean values ​​and standard deviation. The Shapiro-Wilk normality test analyzed the normal distribution of the data, and differences among the groups were determined using the Analysis of Variance (ANOVA) test, considering a significance level of 5% (*p*<0.05). The t-test was also applied to a sample to evaluate the variation in mouth opening and edema after 7 days, separately in each group.

## Results

**Figure 1:**
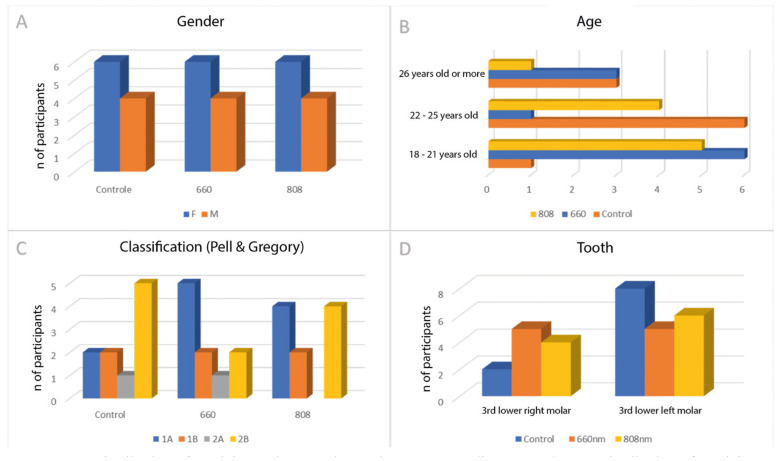
A. Distribution of participants by experimental groups according to gender; B. Distribution of participants by experimental groups according to age; C. Distribution of participants by experimental groups according to Pell and Gregory's classification for impacted third molars; D. Distribution of dental elements extracted by experimental groups.

A total of 33 patients were included, considering a margin of 10% of dropouts. Then, after the equal and randomized distribution of the patients, 33 surgeries were performed. Three participants dropped out due to not returning for postoperative evaluations, as previously combined, and thus were excluded from the project. The results are demonstrated below (Fig. 1).

**Figure 2:**
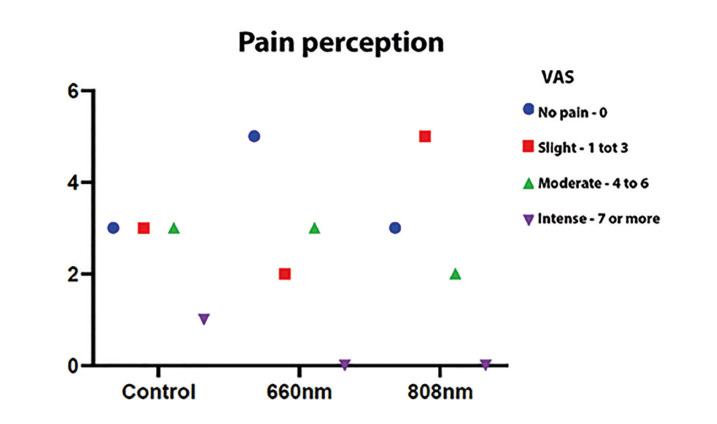
The mean and standard deviation, according to the VAS, for the perception of postoperative pain after 7 days.

**Figure 3:**
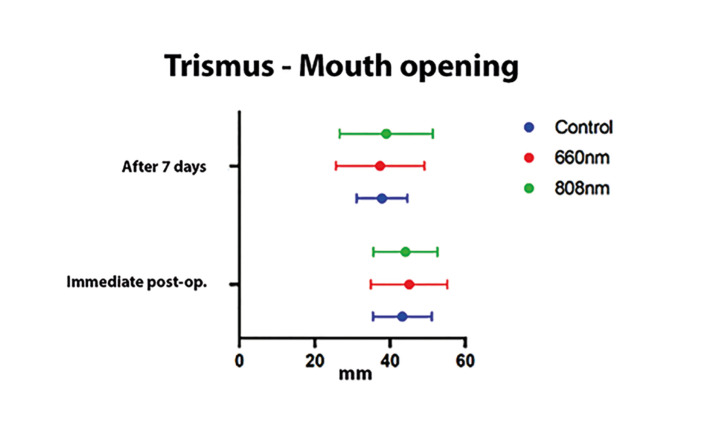
The mean and standard deviation for mouth opening parameter in millimeters among the different experimental groups (Control, 660 nm, and 808 nm) immediately and after 7 days. After the (Shapiro-Wilk) normality test, the groups were subjected to statistical analysis (ANOVA) to evaluate differences in the respective experimental periods (*p*<0.05).

**Figure 4:**
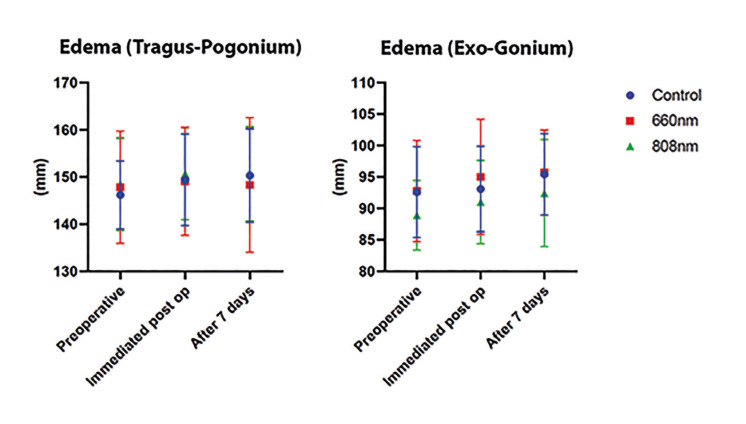
The mean and standard deviation of the volume of edema measured from pre-determined facial points before, immediately at the end of the procedure, and after 7 days of the surgery.

The mean and standard deviation according to the VAS were registered, referring to the perception of postoperative pain of participants undergoing third molar extraction and included in the different experimental groups (Control, 660 nm, and 808 nm) after seven days (Fig. 2); the results showed no statistically significant differences in postoperative pain after seven days between the groups studied (*p*>0.05). The mean and standard deviation for mouth opening parameter among the different experimental groups (Control, 660 nm, and 808 nm), immediately and after seven days (Fig. 3), presented a significant difference between the experimental periods for the control group (*p*=0.042) and group 2 (*p*=0.039) - intragroup analysis; otherwise, there were no statistically significant differences in mouth opening after 7 days between the groups studied (*p*>0.05). The mean and standard deviation for the volume of edema measured, from pre-determined facial points, before, immediately at the end of the procedure, and after 7 days of the surgery presented no statistically significant differences between the different groups and periods studied (Fig. 4).

## Discussion

The present study aimed to evaluate and discuss the effects of using Low Power Laser (LLLT) with wave frequencies of 660nm and 808nm on pain, edema, and difficulty in opening the mouth observed in the postoperative period of patients undergoing surgery for the extraction of impacted lower third molars, seeking to establish the best therapeutic protocol. Scientific evidence indicates that the LLLT has analgesic, anti-inflammatory, and biostimulant effects and increases tissue nutrition and drainage through the lymphatic system (15,19). These effects beneficially control pain, edema, and trismus (17). However, the application can vary according to the LLLT, such as the type of laser, wavelength applied, and lasting time, which are still controversial (5). In this sense, randomized clinical studies have been conducted to evaluate the effectiveness of LLLT in postoperative control, in addition to standardizing protocols (20-25).

He *et al*. (26), through a systematic review of randomized clinical studies, evaluated the effectiveness of LLLT in reducing complications caused by lower third molar extractions. In the studies analyzed in comparison with the control group (laser-placebo), the LLLT group significantly reduced pain on the first, second, and third postoperative days (*p*<0.00001). Furthermore, LLLT showed beneficial effects in lowering trismus and edema after 3 and 7 days of tooth extraction with intra and extraoral applications. Despite the favorable results, due to the heterogeneity observed for the interventions, the authors suggested that new clinical studies with larger samples are necessary to expand the scientific evidence about LLLT (26).

Three studies based on the abovementioned SR (26) were carried out. Sierra *et al*. (27), in a randomized clinical study, investigated five different application techniques associated with various types of wavelengths: 660 nm/intraoral application, 660 nm/extraoral application, 808/intraoral application, 808/extraoral application, and controls (intraoral and extraoral placebo). The authors concluded that a single treatment session with LLLT did not significantly reduce pain after extraction of mandibular third molars under the conditions investigated. Regarding the use of multiple applications of LLLT after extraction of third molars, Pol *et al*. (28) presented indicative results of pain and edema reduction with a protocol of three applications in the immediate post-operative period, after 24 and 28 hours of the procedure with a wavelength varying between 904/910 nm. Momeni *et al*. (23), in another randomized clinical trial with 25 participants, showed results that suggested an improvement in pain perception and postoperative trismus and edema. Still, despite this, only pain perception had a significant difference in favor of the irradiated group.

To reduce biases resulting from disparate selections that could distort the results in our study, the dental elements included in the analysis were classified according to the well-known classification developed by Winter and Pell and Gregory, respectively, considering the inclination of the tooth, in addition to its relationship with the mandibular anterior edge and the occlusal plane. In the present study, of the 30 participants who completed the follow-up, there was a prevalence of females in all groups, ranging from 18 to 36 years. Of these, only 7 participants were aged 26 or over, confirming expectations regarding the most expected age profile. As for the lower third molars, in our sample, 19 were on the left side while 11 were on the right side, and it was noticed that after randomization, in the control group, type 2B teeth prevailed according to the Pell & Gregory classification, which could perhaps justify some of the results found.

Regarding the assessment of pain perception, the method adopted efficiently allowed to verify that, unlike the control group, in the experimental groups 1 and 2, there was no report of intense pain after seven days postoperatively. Although there was no statistically significant difference when comparing the three groups, the results suggested, in agreement with previous studies (23,24), lower perception of pain in irradiated participants, especially those in group 1 (660nm).

Regarding the assessment of postoperative trismus, when comparing the individual mouth opening variation in each group, it was observed that only group 1 (660 nm) did not show a significant difference between immediate mouth opening and after seven days. However, when comparing the groups, the results showed no statistically significant difference. Likewise, there was no statistically significant difference between the experimental groups immediately after the surgeries or after seven days, either in evaluating the dimensions between the exocantium-gonium or tragus-pogonium faciometric points.

## Conclusions

Thus, despite the results showing no differences between the groups, using LLLT resulted in lower pain perception in the irradiated participants compared to those in the control group. This fact supports the use of LLLT as an adjuvant therapy in patients undergoing oral surgery procedures to remove impacted third molars. Therefore, new studies should be carried out in order to establish the best use of this therapeutic resource.
